# Early arrhythmia recurrence during the blanking period after pulsed-field ablation for atrial fibrillation

**DOI:** 10.1016/j.hroo.2026.05.012

**Published:** 2026-06-05

**Authors:** Fabian Jordan, Corinne Isenegger, Bedran Peker, Jonas Brügger, Sven Knecht, Philipp Krisai, Nicolas Schaerli, Gian Völlmin, Reto Stump, David Spreen, Felix Mahfoud, Christian Sticherling, Michael Kühne, Patrick Badertscher

**Affiliations:** 1 Department of Cardiology, University Hospital Basel, Basel, Switzerland; 2 Cardiovascular Research Institute Basel, University Hospital Basel, Basel, Switzerland

**Keywords:** Atrial fibrillation, Pulsed-field ablation, Pulmonary vein isolation, Arrhythmia recurrence, Blanking period, Outcomes, Kaplan-Meier

## Abstract

**Background:**

Pulmonary vein isolation is a cornerstone in the treatment of atrial fibrillation, with procedural success traditionally assessed after a 3-month “blanking period” to account for early postablation arrhythmias. However, emerging evidence challenges this paradigm, particularly with nonthermal energy modalities such as pulsed-field ablation (PFA), which induce minimal inflammatory response.

**Objective:**

The purpose of this study was to assess the prognostic relevance of early recurrence (ER) of atrial arrhythmia during the blanking period after PFA-based pulmonary vein isolation, with a focus on the timing of ER and its association with late recurrence (LR).

**Methods:**

In this single-center study, 241 patients with atrial fibrillation underwent first-time PFA and were followed for ≥12 months. *ER* was defined as any atrial arrhythmia lasting >30 seconds within the first 3 months of ablation. The primary outcome was freedom from LR beyond the blanking period.

**Results:**

ER occurred in 48 (20%) of patients, with 23 (48%) occurring within the first month and 25 (52%) during the second or third month of ablation. LR was significantly more frequent in patients with ER (*P* < .001), particularly when recurrence occurred after day 30. Multivariable Cox analysis identified ER during the second or third month (hazard ratio 6.1; 95% confidence interval 3.3–11.3) and ER during the first month (hazard ratio 3.6; 95% confidence interval 1.8–7.1) as the strongest independent predictors of LR. A subset of ERs resolved spontaneously.

**Conclusion:**

The timing of ER after PFA strongly predicts LR, supporting reconsideration of the 90-day blanking period. It may be appropriate to shorten the blanking period for PFA, but late ER warrants clinical attention. Larger studies should validate timing-based blanking strategies.

**Key Findings**

▪ The timing of early recurrence after pulsed-field ablation is independently associated with late arrhythmia recurrence, with later events in the blanking period carrying greater prognostic significance.
▪ Early recurrences occur predominantly within the first month and decrease over time, suggesting a temporal pattern influenced by transient procedural effects.
▪ Early recurrence likely reflects both procedural factors and underlying atrial substrate vulnerability rather than a purely procedural phenomenon.

## Introduction

Pulmonary vein isolation (PVI) is a foundational therapy for atrial fibrillation (AF), with procedural success traditionally evaluated after a 3-month “blanking period.”^1^ This concept, introduced in 2007, was intended to account for early, often transient, arrhythmias after ablation typically attributed to inflammatory changes caused by thermal energy. During this period, arrhythmia recurrences have generally not been considered indicative of treatment failure.^2^ However, emerging evidence has challenged this assumption, revealing a substantial proportion of early recurrences (ERs) to be predictive of late recurrences (LRs), prompting expert groups to reconsider the duration of the blanking period.^3^, ^4^, ^5^, ^6^, ^7^, ^8^, ^9^ Notably, the 2024 European Heart Rhythm Association consensus document now recommends shortening the blanking period to 8 weeks after thermal AF ablation.^10^

Pulsed-field ablation (PFA) is a novel nonthermal ablation technique that uses high-voltage, short-duration electrical pulses to achieve myocardial tissue ablation through irreversible electroporation. Compared with thermal modalities such as radiofrequency or cryoablation, PFA appears to elicit a milder and shorter-lived inflammatory response, with histological evidence suggesting resolution within 30 days.^11^^,^^12^ These mechanistic differences raise an important clinical question: whether the traditional 3-month blanking period remains applicable in the context of PFA.

Accordingly, the objective of this study was to evaluate the prognostic significance of early arrhythmia recurrence within the conventional 3-month blanking period in patients undergoing PVI with PFA, with a particular focus on the impact of ER timing on the risk of subsequent LR.

## Methods

### Patient population

This single-center study retrospectively analyzed prospectively enrolled patients from the Swiss Atrial Fibrillation Pulmonary Vein Isolation Registry (ClinicalTrials.gov identifier NCT03718364) who underwent their first PVI using PFA from January 2022 to January 2024 and were followed for at least 365 days. Exclusion criteria were patients with incomplete follow-up. Written informed consent and age ≥ 18 years were mandatory. The study was performed according to the principles of the Declaration of Helsinki and was approved by the local ethics committees.

### Procedures

#### Preprocedural management

All patients underwent preprocedural transesophageal echocardiography to rule out left atrial thrombus. In addition, all patients underwent preprocedural imaging with either computed tomography or magnetic resonance imaging of the left atrium.

#### Access

Midazolam, fentanyl, and propofol were used for sedation. Femoral access was obtained using ultrasound guidance in all cases. The transseptal puncture was performed via fluoroscopic guidance or in selected cases using intracardiac echocardiography. Intravenous heparin was used to keep the activated clotting time at a target value of 350 seconds. The intracardiac electrograms and surface electrograms were recorded at a speed of 100 mm/s (Sensis, Siemens, Erlangen, Germany). No contrast was used during the procedure.

#### PFA

For PFA, the FARAPULSE Pulsed Field Ablation System (Boston Scientific, Natick, MA) was used. After transseptal puncture, a J-tip guidewire was used to cannulate the pulmonary vein (PV) and the device was deployed inside the left atrium.

The PFA procedure was performed using a standard protocol,^13^^,^^14^ with 4 applications in basket configuration and 4 applications in flower configuration per vein (at least 32 applications). To ensure uniform coverage across the entire circumference, a rotation of the catheter by 30°–40° was carried out after 2 successive applications in each configuration. Additional lesions for the right-sided carina as well as for non-PV triggers such as the posterior wall were applied at the discretion of the operator. Ablation procedures were performed using 2 kV.

The *procedural end point* was defined as PVI, assessed directly at the end of the procedure for all PVs. For end point confirmation, entrance and, if deemed necessary, exit block were tested via the PFA catheter by pacing with 10 V and a pulse width of 1.5 ms among all electrodes or via a 3-dimensional electroanatomic mapping system (CARTO 3, Biosense Webster, Irvine, CA).

#### Postprocedural management

Oral anticoagulation continued for at least 2 months after ablation. Hemostasis was established using a figure-of-eight sutures, followed by 4 hours of bed rest. A pericardial effusion was ruled out by transthoracic echocardiography within 1 hour of intervention.

Antiarrhythmic drugs were in general continued in patients with persistent AF who had tolerated antiarrhythmic medication before ablation. In others who experienced antiarrhythmic drug–related adverse effects, antiarrhythmic drugs were discontinued. Discontinuation of β-blocker therapy was at the discretion of the treating physician. In general, β-blocker therapy was discontinued after ablation in patients with paroxysmal AF and if AF was the sole indication for β-blocker therapy.

### Recurrence definitions and follow-up

Any atrial arrhythmia, including AF, atrial flutter, and atrial tachycardia, lasting >30 seconds was considered a recurrence. The *blanking period* was defined as the 3 months after the ablation procedure. *ER* referred to any atrial arrhythmia occurring within the blanking period. *LR* was defined as any recurrence occurring beyond the 3-month blanking period. *Resolved recurrence* was defined as the absence of LR at 12-month follow-up in patients who had previously experienced ER. *Spontaneous recurrences* were defined as atrial arrhythmias that stopped without therapeutic intervention.

All patients underwent follow-up consisting of outpatient clinic visits after 3, 6, and 12 months, including physical examination, 12-lead electrocardiogram (ECG), and 7-day Holter ECG. In case of symptomatic recurrence outside scheduled follow-up, either a 12-lead ECG or a Holter ECG was recorded for arrhythmia documentation. Patients participating in the PIFPAF-AF or SINGLE SHOT CHAMPION study (ClinicalTrials.gov identifiers NCT05986526 and NCT05534581) received an implantable loop recorder (ILR). Apart from this, clinical workup, procedural approach, and postprocedural management were identical regardless of study participation. In patients undergoing repeat catheter ablation, a voltage map of the left atrium was created using a 3-dimensional electroanatomic mapping system (CARTO 3) and generated with a multipolar mapping catheter (PENTARAY or OCTARAY, Biosense Webster).

### Statistical analysis

Continuous variables are reported as median and interquartile range (IQR), except for CHA_2_DS_2_-VA score, which was reported as mean and standard deviation, and compared using the Wilcoxon rank sum test. Categorical variables are presented as numbers and percentages and compared using the χ^2^ or Fisher exact test, as appropriate. The Kaplan-Meier analysis with a log-rank test was used to compare the probability of freedom from the LR of atrial arrhythmia. A *P* value <.05 was considered statistically significant. A multivariable Cox proportional hazards model was fitted to identify predictors of LR. Potential confounders were entered into the model on the basis of known or expected clinical relevance, regardless of statistically significant differences in the populations. Variables included in the model were age, BMI, sex, CHA_2_DS_2_-VA score without age accounted to reduce interactions between this variable and age (CHA_2_DS_2_-VA score without age), AF type, additional lesions, presence of left common PV, and ILR implantation. Hazard ratios (HRs) along with 95% confidence intervals (CIs) from the Cox proportional hazards model are reported in the Results section. Analysis was performed using R (R Core Team [2021], R Foundation for Statistical Computing, Vienna, Austria) and RStudio 2023.09.1 (RStudio Team [2019], RStudio, Inc., Boston, MA).

## Results

### Baseline characteristics

A total of 241 consecutive patients were included (median age of 67 years; IQR 59–74 years; 33.6% female). ER was observed in 48 patients (20%), while 193 patients (80%) remained free of ER during the blanking period. Persistent AF was present in 118 patients (49%). The median left atrial diameter was 41 mm (IQR 37–46 mm) in the whole population and was similar between patients with and without ER (41 mm [IQR 37–46 mm] with ER vs 41 mm [IQR 38–45 mm] without ER; *P* = .843). The median left ventricular ejection fraction was 55% (IQR 50%–60%) in the ER group vs 58% (IQR 50%–63%) in the no ER group, with no statistically significant difference between the groups (*P* = .117). ILR implantation was not significantly different between patients with and without ER (10 [20.8%] vs 25 [13.0%]; *P* = .247). Baseline characteristics are summarized in Table 1.

**Table 1:** Baseline characteristics

Characteristic	Overall	No early recurrence	Early recurrence	*P*
	(N = 241)	(n = 193)	(n = 48)	
Female	81 (33.6)	63 (32.6)	18 (37.5)	.641
Age (y)	67 (59–74)	66 (59–73)	69 (62–74)	.288
BMI (kg/m^2^)	26.6 (23.9–29.4)	26.6 (23.7–29.4)	26.9 (24.6–31.2)	.447
CHA_2_DS_2_-VA score	1.96 ± 1.38	1.84 ± 1.32	2.44 ± 1.53	.007
Years from diagnosis to PFA	1.63 (0.46–4.85)	1.46 (0.46–4.78)	2.32 (0.53–5.56)	.329
Persistent AF	118 (49.0)	92 (47.7)	26 (54.2)	.519
Diabetes	23 (9.5)	17 (8.8)	6 (12.5)	.614
Hypertension	140 (58.1)	111 (57.5)	29 (60.4)	.84
Hypercholesterolemia	90 (37.3)	59 (30.6)	31 (64.6)	<.001
CAD	28 (11.6)	16 (8.3)	12 (25.0)	.003
Heart failure	43 (17.8)	34 (17.6)	9 (18.8)	.99
OSAS	25 (10.4)	18 (9.3)	7 (14.6)	.421
Stroke	3 (1.2)	1 (0.5)	2 (4.2)	.189
Thyroid dysfunction	22 (9.1)	15 (7.8)	7 (14.6)	.236
Smoking history	128 (53.1)	92 (47.7)	36 (75.0)	.001
LCPV	36 (14.9)	26 (13.5)	10 (20.8)	.292
LAD (mm)	41 (37–46)	41 (37–45)	41 (38–46)	.843
LAVI (mL/m^2^)	39 (30–47)	39 (30–46)	41 (33–51)	.295
LVEF (%)	57 (50–63)	58 (50–63)	55 (50–60)	.117
AAD	99 (41.1)	77 (39.9)	22 (45.8)	.577
Class I AAD	31 (12.9)	24 (12.4)	7 (14.6)	.875
Class III AAD	72 (29.9)	57 (29.5)	15 (31.2)	.955
Digoxin	3 (1.2)	1 (0.5)	2 (4.2)	.189
Verapamil	2 (0.8)	0 (0)	2 (4.2)	.05
β-Blocker	172 (71.4)	135 (69.9)	37 (77.1)	.424
Vitamin K antagonist (ie, Phenprocoumon)	10 (4.1)	7 (3.6)	3 (6.3)	.745
DOAC	231 (95.9)	186 (96.4)	45 (93.7)	.745
ILR	35 (14.5)	25 (13.0)	10 (20.8)	.247

Continuous variables are presented as median (interquartile range), except for CHA_2_DS_2_-VA score, which is presented as mean ± standard deviation, and factorial variables as n (%).

Early recurrence: atrial arrhythmia of >30-s duration during the first 3 mo postablation.

AAD = antiarrhythmic drug; AF = atrial fibrillation; BMI = body mass index; CAD = coronary artery disease; DOAC = direct oral anticoagulation; ILR = implantable loop recorder; LAD = left atrial diameter; LAVI = left atrial volume index; LCPV = left common pulmonary vein; LVEF = left ventricular ejection fraction; OSAS = obstructive sleep apnea syndrome; PFA = pulsed-filed ablation.

### Procedural characteristics

Acute PVI success was achieved in 239 patients (99%). Procedural characteristics were comparable between patients with and without ER (Table 2).

**Table 2:** Procedural characteristics

Characteristic	Overall	No early recurrence	Early recurrence	*P*
	(N = 241)	(n = 193)	(n = 48)	
Procedure time (min)	55 (41–69)	54 (41–68)	58 (42–69)	.574
LA dwell time (min)	37 (26–52)	37 (26–53)	38 (28–50)	.919
Fluoroscopy time (min)	11 (9–14)	11 (8–14)	11 (9–15)	.160
Fluoroscopy dose (μGy⋅m^2^)	451 (254–929)	434 (251–929)	465 (310–923)	.620
Total applications	32 (27–40)	32 (26–40)	32 (28–42)	.697
Additional lesions	71 (29.5)	58 (30.1)	13 (27.1)	.821
CTI	27 (11.2)	23 (11.9)	4 (8.3)	.799
Basket applications	16 (8–16)	16 (8–16)	16 (8–16)	.917
Flower applications	16 (10–18)	16 (10–18)	16 (10–18)	.550
Catheter size 35 mm	10 (4.1)	10 (5.2)	0 (0)	.228

Continuous variables are presented as median (interquartile range) and factorial variables as n (%).

CTI = cavotricuspid isthmus; LA = left atrial.

The median procedure duration was 55 minutes (IQR 41–69 minutes) (58 minutes [IQR 42–69 minutes] in the ER group vs 54 minutes [IQR 41–68 minutes] in the no ER group; *P* = .574). The left atrial dwell time and fluoroscopy time were 38 minutes (IQR 28–50 minutes) and 11 minutes (IQR 9–15 minutes) in the ER group vs 37 minutes (IQR 26–53 minutes) and 11 minutes (IQR 8–14 minutes) in the no ER group (*P* = .919 and *P* = .160, respectively).

PVI only was performed in 170 patients (70.5%), while the remaining 71 patients (29.5%) underwent additional ablation procedures targeting non-PV triggers, mostly posterior wall isolation. Among them, 27 patients (11.2%) also underwent cavotricuspid isthmus ablation. The median number of pulsed-field applications was 32 (IQR 27–40). There was no significant difference between ER and no ER (32 [IQR 28–42] vs 32 [IQR 26–40]; *P* = .697).

### Procedural safety

A total of 4 complications (1.7%) were observed. Pericardial tamponade occurred in 2 patients (0.8%) and was successfully treated with drainage. 1 patient experienced an ischemic stroke without a residual neurological deficit. 1 patient experienced a sinus arrest with transient ST-segment elevation; subsequent coronary angiography showed no stenosis or spasm.

Both patients with tamponade experienced ER within the first month, which resolved spontaneously without treatment, and none of the patients with complications had LR during the first year of follow-up.

#### ER

Among the 48 patients with ER, 23 patients (48%) had episodes during the first month, 9 patients (19%) during the second month, and 16 patients (33%) during the third month. Recurrences were detected by ILRs in 6 first month cases (26%), 0 second month cases (0%), and 4 third month cases (25%), without clustering in later periods.

Among patients with ER during the first month (n = 23), recurrence resolved in 10 patients (43%), with 4 (40%) converting spontaneously. The remaining 6 patients (60%) had their antiarrhythmic drug therapy escalated (2 [33%] with β-blocker and 4 [67%] with amiodarone), and in 2 patients (33%), electrical cardioversion was performed. In the group with recurrence during the second or third month (n = 25), recurrence resolved in 6 patients (24%), including 3 (50%) with spontaneous conversion. The remaining 3 patients (50%) required electrical cardioversion; 2 patients (33%) had their antiarrhythmic drug therapy escalated with amiodarone.

Accordingly, LR postablation was observed in 13 patients (57%) with ER during the first month and in 18 patients (72%) with ER during the second or third month. An overview regarding ER is provided in Table 3.

**Table 3:** Early recurrences

	Overall (N = 48)	Early recurrence at the first month		Early recurrence at the second or third month	
		Resolved	Late recurrence	Resolved	Late recurrence
		(n = 10)	(n = 13)	(n = 6)	(n = 19)
Early recurrence arrhythmia					
AF	36 (75)	6 (60)	12 (92)	6 (100)	12 (63)
AT	6 (12.5)	1 (10)	1 (8)	0 (0)	4 (21)
Atrial flutter	6 (12.5)	3 (30)	0 (0)	0 (0)	3 (16)
Spontaneous resolved∗	11 (23)	4 (40)	3 (23)	3 (50)	1 (5)
AAD therapy added/escalated	20 (42)	6 (60)	5 (38)	2 (33)	7 (37)
β-Blocker	7 (35)	2 (33)	1 (20)	0 (0)	4 (57)
Amiodarone	13 (65)	4 (67)	4 (80)	2 (100)	3 (43)
Electrical cardioversion	21 (44)	2 (20)	7 (54)	3 (50)	9 (47)
Repeat catheter ablation†	16 (33)	0 (0)	6 (46)	0 (0)	13 (68)

Factorial variables are presented as n (%).

AAD = antiarrhythmic drug; AF = atrial fibrillation; AT = atrial tachycardia;.

∗ Spontaneous resolved: early recurrence disappeared without therapeutic intervention, such as electrical cardioversion or escalation of antiarrhythmic therapy.

† Repeat catheter ablation: additional electrophysiology study to treat late recurrence.

#### LR

All patients completed at least 1 year of follow-up. Among the 241 included patients, 35 (15%) had an ILR (Supplemental Tables 1 and 2): 10 (21%) with ER and 25 (13%) without ER (*P* = .247).

**Figure 1:**
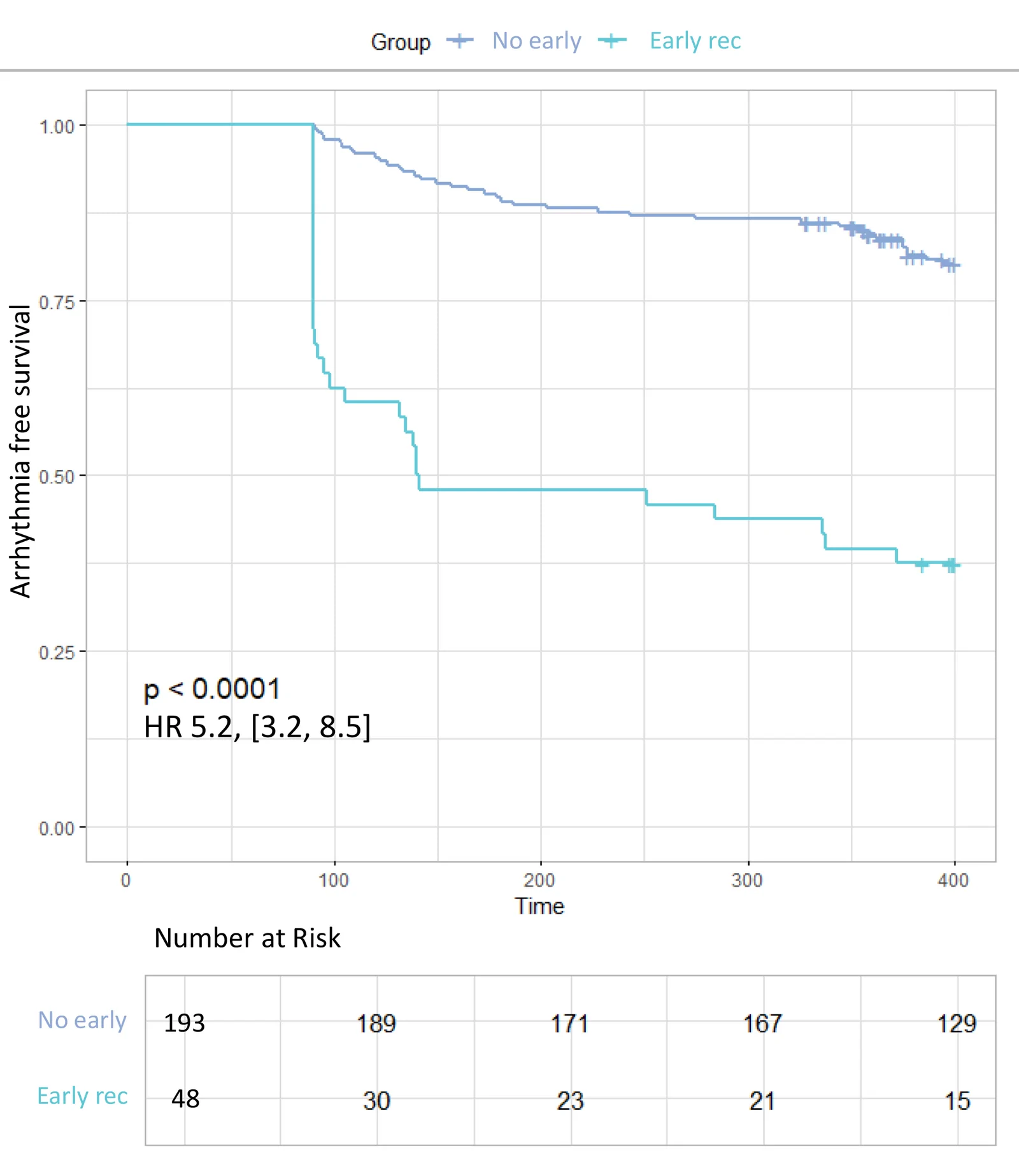
Kaplan-Meier curve comparing the time to rearrhythmia of patients with (Early rec) and without (No early) early recurrence. The log-rank test was used to determine the *P* value. Time in days. Hazard ratio (HR) 'Early rec' to 'No early'.

**Figure 2:**
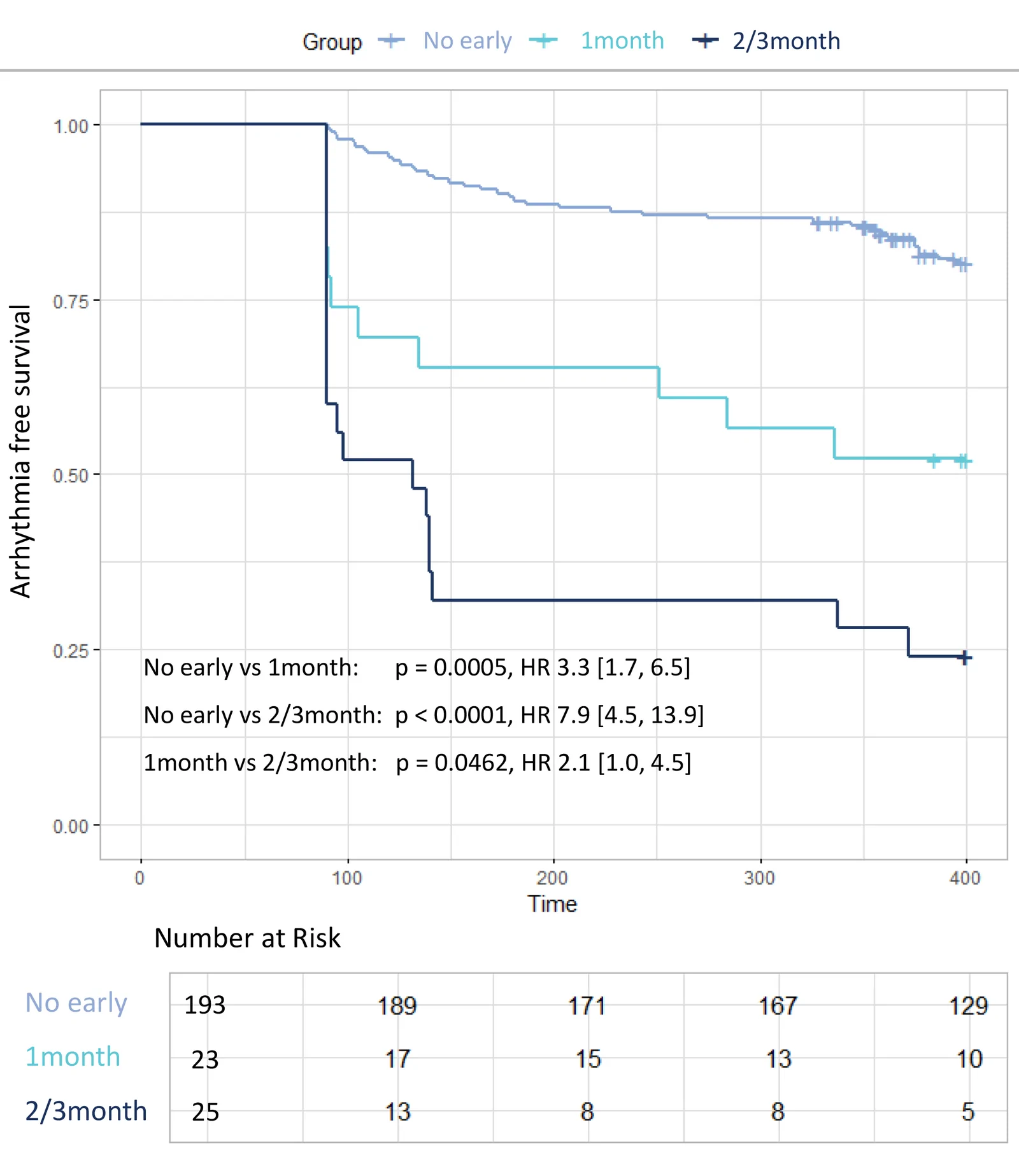
Kaplan-Meier curve comparing the time to rearrhythmia of patients without early recurrence (No early), patients with early recurrence in the first month (1 month), and patients with early recurrence in the second or third month (2/3 month). The log-rank test was used to determine the *P* value. Time in days. HR = hazard ratio.

Overall, 75 (31%) experienced LR, of whom 60 patients (80%) did so within the first year of ablation, including 29 patients (60%) with ER and 31 patients (16%) without ER (Table 4). This resulted in a 1-year recurrence-free survival rate of 75% (n = 181). The success rate was significantly higher in patients without ER, with a rate of 84% (n = 162) compared with 40% (n = 19) in those with ER (HR for LR 5.2; 95% CI 3.2–8.5; *P*_Log-rank_ < .0001) (Figure 1). Similar findings were observed in subgroup analyses: patients with recurrence during the first month had a significantly lower 1-year recurrence-free survival than did those without ER (52% (n = 12) vs 84% (n = 162); HR for LR 3.3; 95% CI 1.7–6.5; *P*_Log-rank_ = .0005), and this association was even more prominent in patients with recurrence during the second or third month (28% (n = 7) vs 84% (n = 162); HR for LR 7.9; 95% CI 4.5–13.9; *P*_Log-rank_ < .0001). In addition, the incidence of LR after 1 year was 52% (n = 12) in patients with ER in the first month vs 28% (n = 7) in those with ER in the second or third month (HR for LR 2.1; 95% CI 1.0–4.5; *P*_Log-rank_ = .0462; Figure 2).

**Table 4:** Late recurrences in the first year postablation

	Overall	No early recurrence	Early recurrence
	(N = 60)	(n = 31)	(n = 29)
Recurrence arrhythmia			
AF	53 (88)	30 (97)	23 (79)
AT	5 (8)	1 (3)	4 (14)
Atrial flutter	4 (7)	1 (3)	3 (10)
Spontaneous resolved∗	12 (20)	9 (29)	3 (10)
AAD therapy added/escalated	18 (30)	8 (26)	10 (34)
β-Blocker	9 (50)	3 (38)	6 (60)
Amiodarone	9 (50)	5 (62)	4 (40)
Electrical cardioversion	11 (18)	5 (16)	6 (21)
Ablation (EPS-RFA/AV node ablation)	6 (10)	2 (6)	4 (14)
Repeat catheter ablation†	34 (57)	16 (52)	18 (62)

Factorial variables are presented as n (%).

AAD = antiarrhythmic drug; AF = atrial fibrillation; AT = atrial tachycardia; EPS-RFA/AV node ablation = electrophysiology study with radiofrequency ablation or atrioventricular node ablation.

∗ Spontaneous resolved: late recurrence disappeared without therapeutic intervention, such as electrical cardioversion or escalation of antiarrhythmic therapy.

† Repeat catheter ablation: additional electrophysiology study to treat late recurrence.

The redo rate among patients with LR after 1 year was 57% (n = 34). This did not differ statistically significantly between patients with ER and those without ER (18 [62%] vs 16 [52%]; *P* = .578).

### Redo procedures

Overall, 41 patients (17%) underwent a redo procedure, of whom 7 underwent a redo procedure because of LR after more than a year: 19 patients (40%) in the ER group and 22 patients (11%) in the no ER group (*P* < .001). For details on redo procedures, see Table 5.

**Table 5:** Redo procedures

	Overall	No early recurrence	Early recurrence
	(N = 41)	(n = 22)	(n = 19)
Days to redo procedures	224 (168–352)	286 (213–433)	185 (148–232)
No. of reconnected veins			
0	19 (46.3)	10 (45.5)	9 (47.4)
1	12 (29.3)	7 (31.8)	5 (26.3)
2	7 (17.1)	3 (13.6)	4 (21.1)
3	1 (2.4)	0 (0)	1 (5.3)
4	2 (4.9)	2 (9.1)	0 (0)
Reconnected veins			
RSPV	13 (31.7)	7 (31.8)	6 (31.6)
LSPV	10 (24.4)	7 (31.8)	3 (15.8)
RIPV	8 (19.5)	3 (13.6)	5 (26.3)
LIPV	6 (14.6)	4 (18.2)	2 (10.5)

Continuous variables are presented as median (interquartile range) and factorial variables as n (%).

LIPV = left inferior pulmonary vein; LSPV = left superior pulmonary vein; RIPV = right inferior pulmonary vein; RSPV = right superior pulmonary vein.

Among patients with ER within the first month, 6 of 23 (26%) underwent redo ablation. In those with recurrence during the second and third months, 13 of 25 (52%) underwent redo procedures, including 6 of 9 patients (67%) with recurrence in the second month and 7 of 16 patients (44%) with recurrence in the third month.

Regarding PV durability, no significant differences were observed between patients with and without ER, nor between those with recurrence in the first month and those with recurrence in the second or third month.

The rate of patients who underwent a redo procedure for LR after 1 year was not statistically significant different, irrespective of whether ER occurred during the first month or the second or third month (6 of 11 [55%] vs 12 of 18 [67%]; *P* = .796). Repeat ablation procedures were performed at a median of 224 days (IQR 168–352 days) after the initial procedure.

**Figure 3:**
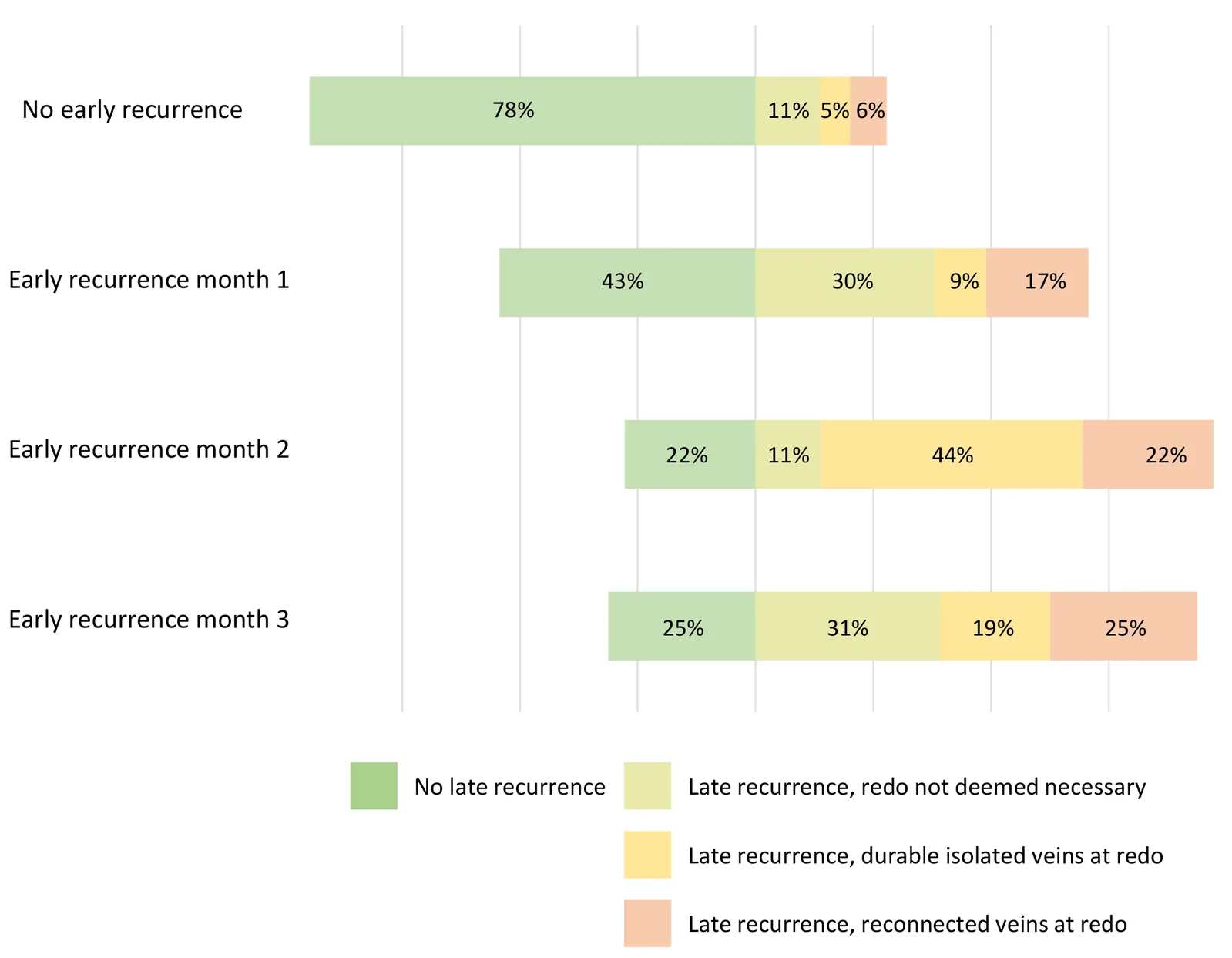
Mirror bar plot with percentages of groups on the x-axis.

A summary of LR, redo catheter ablation, and durably isolated veins is presented in Figure 3.

### Predictors of LR in multivariable analysis

**Figure 4:**
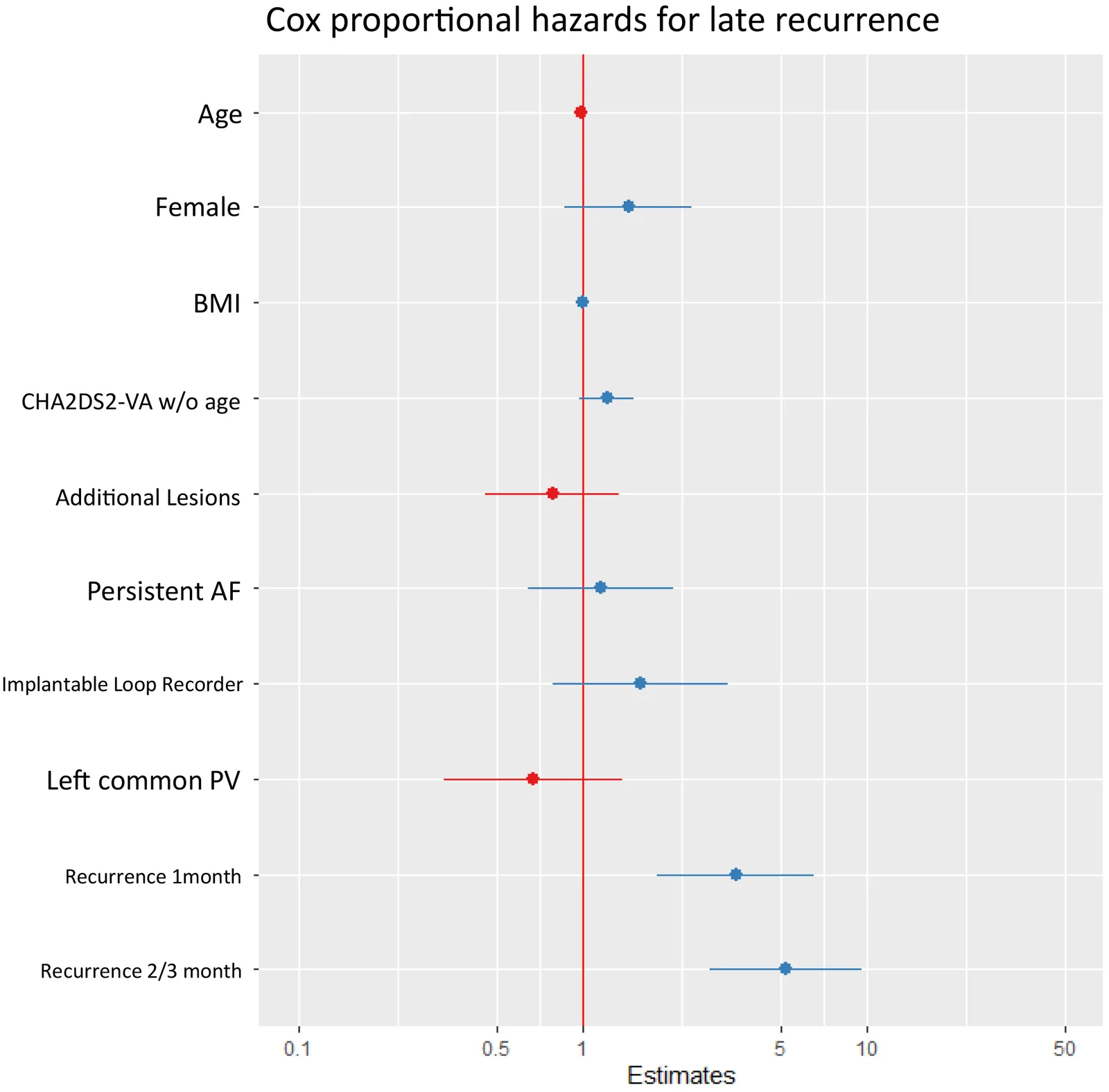
Cox proportional hazards model for late recurrence with hazard ratios. CHADS_2_VA score was treated as a numeric variable as it was approximately normally distributed. For the exact numbers, please see Table S1. AF = atrial fibrillation; BMI = body mass index; CHADS_2_VA = chronic heart failure, hypertension, age ≥ 75 years, diabetes, stroke/transient ischemic attack/thromboembolism, vascular disease, age 65-74 years; PV, pulmonary vein.

In a multivariable Cox proportional hazards model, adjusting for age, BMI, sex, CHA_2_DS_2_-VA score, AF type, additional lesions, presence of left common PV, and ILR implantation, the only independent predictors of LR were recurrence at the first month (HR 3.53; 95% CI 1.76–7.05) and at the second or third month (HR 6.07; 95% CI 3.25–11.33) (Figure 4 and Supplemental Table 3).

## Discussion

This study assessed the prognostic relevance of ER of atrial tachyarrhythmias during the blanking period after PFA for AF. Our findings reveal several key insights with implications for clinical practice.

First, ER occurred in ∼20% of patients, with half of events arising within the first month. Importantly, the timing of ER, particularly in the second and third months, emerged as a stronger predictor of LR than type of AF or mode of follow-up. While 40%–45% of ERs in the first month stayed free of arrhythmia once it was resolved, this was true for only less than a quarter of those occurring later, underscoring a temporal gradient in prognostic significance.

ER may reflect not only procedural factors such as lesion maturation and transient inflammation but also underlying atrial substrate vulnerability. Patients with ER in our cohort exhibited a higher burden of cardiovascular risk factors, suggesting more advanced atrial disease that may predispose to both early and late arrhythmia recurrences. The observed temporal gradient is therefore likely multifactorial, with ER representing a combination of transient procedural effects and a marker of intrinsic substrate susceptibility.

These data align with prior findings from Mohanty et al^9^ and Boersma et al,^15^ who explored ER timing after PFA. While Mohanty et al^9^ reported that no ER in the second or third month resolved, Boersma et al^15^ found that a proportion, particularly in the second month, did not translate into LR. Our findings support a more nuanced interpretation: while late ER strongly predicts recurrence, a subset of patients may still achieve durable rhythm control even after events near the end of the blanking period.

Compared with thermal ablation modalities such as radiofrequency ablation or cryoablation, the overall incidence of ER after PFA appears lower. Previous studies have reported ER in 31%–56% of patients undergoing radiofrequency ablation^3^^,^^5^^,^^8^^,^^16^^,^^17^ compared with 16%–29% after PFA.^9^^,^^15^^,^^18^ This difference likely reflects the nonthermal mechanism of PFA. Irreversible electroporation seems to have a minimal inflammatory response. Preclinical studies have demonstrated that PFA lesions are more homogenous and exhibit less immune cell infiltration than thermal lesions.^19^^,^^20^ Inflammation after PFA resolves within 30 days,^19^ supporting a narrower window for transient arrhythmias and reinforcing our observation that most ER clustered early and declined steeply thereafter (Supplemental Figure 1).

The 2024 European Heart Rhythm Association consensus document^10^ recommends shortening the blanking period from 90 days to 8 weeks after thermal ablation. Our results extend this rationale to PFA: ERs after day 60 were rare but, when present, correlated strongly with LRs. However, shortening the blanking period must be balanced against the risk of overdiagnosing procedural failure. In our cohort, a small number of patients with ER between days 60 and 90 remained arrhythmia-free long term, suggesting that rigid application of an 8-week threshold could prompt unnecessary reintervention in selected cases.

Another noteworthy finding is the lack of association between additional ablation lesions and reduced risk of LR. This contrasts with observations from Mohanty et al,^9^ who reported lower recurrence with more extensive lesion sets. Our multivariable analysis identified only ER timing as an independent predictor of LR.

Finally, the current binary definition of recurrence, based on episodes >30 seconds, may lack granularity. Studies such as De Becker et al^17^ have shown that AF burden during the blanking period is a more informative predictor of long-term outcomes. Future studies using continuous monitoring could redefine treatment success using metrics such as AF burden, episode duration, and temporal clustering of arrhythmia episodes rather than dichotomous recurrence alone.^17^

### Limitations

Several limitations must be acknowledged. First, this was a single-center study, which may limit generalizability. Second, some variables that may be relevant were not accessible in necessary granularity for analysis, such as alcohol consumption, exercise tolerance, blood pressure and glycemic control, obstructive sleep apnea syndrome treatment, thyroid dysfunction management, or cardiorespiratory fitness. Third, no comparison to thermal ablation was made. Although such data were available, this was beyond the scope of this PFA-focused analysis, and differences in patient selection and follow-up would limit interpretability; however, blanking period dynamics after thermal ablation are well established.^12^ Fourth, AF burden was not systematically assessed, as monitoring was mostly limited to intermittent Holter recordings. Nevertheless, follow-up methods reflect routine clinical practice and are consistent with real-world detection of arrhythmia recurrence.^21^^,^^22^ Fifth, nonuniform monitoring may have introduced ascertainment bias, although the distribution of ILR-detected events across blanking period intervals argues against this fully explaining the observed temporal gradient.

## Conclusion

This study supports the relevance of the 8-week blanking period in PFA-treated patients while highlighting the importance of nuanced interpretation of ER, especially those in the third month. A one-size-fits-all approach may not be appropriate. Larger prospective studies with continuous monitoring are needed to validate shortened or dynamic blanking periods tailored to ablation modality and patient-specific risk.

## Disclosures

The authors have no conflicts of interest to disclose.
