# Supplementary material for: Early arrhythmia recurrence during the blanking period after pulsed-field ablation for atrial fibrillation
Source: Heart Rhythm O2. 2026 Jun 5;7(9):1621–30. doi: 10.1016/j.hroo.2026.05.012 (PMC13594461; doi:10.1016/j.hroo.2026.05.012)
Supplement: Supplementary file 1 [file mmc1.docx]

Early Arrhythmia Recurrence during the Blanking Period after Pulsed-field Ablation for Atrial Fibrillation – Supplemental Material

## Table 1 Baseline characteristics ILR

|  | Overall | | Holter | ILR | p |
| --- | --- | --- | --- | --- | --- |
| n | 241 | | 206 | 35 |  |
|  |  | |  |  |  |
| Female | 81 (33.6) | | 73 (35.4) | 8 (22.9) | 0.207 |
| Age | 67 [59, 74] | | 68 [59, 74] | 66 [61, 69] | 0.556 |
| BMI | 26 [23, 29] | 26 [24, 29] | | 25 [23, 28] | 0.075 |
| CHA2DS2-VA | 1.96 (1.38) | | 2.01 (1.42) | 1.66 (1.11) | 0.157 |
|  |  | |  |  |  |
| Years, diagnosis to PFA | 1.63 [0.46, 4.85] | | 1.76 [0.49, 5.07] | 1.09 [0.41, 2.78] | 0.104 |
| Persistent AF | 118 (49.0) | | 117 (56.8) | 1 (2.9) | <0.001 |
| Diabetes | 23 (9.5) | | 20 (9.7) | 3 (8.6) | 1 |
| Hypertonie | 140 (58.1) | | 120 (58.3) | 20 (57.1) | 1 |
| Hypercholesterinemia | 90 (37.3) | | 75 (36.4) | 15 (42.9) | 0.589 |
| CAD | 28 (11.6) | | 23 (11.2) | 5 (14.3) | 0.805 |
| Heart Failure | 43 (17.8) | | 41 (19.9) | 2 (5.7) | 0.074 |
| OSAS | 25 (10.4) | | 21 (10.2) | 4 (11.4) | 1 |
| Stroke | 3 (1.2) | | 3 (1.5) | 0 | 1 |
| Thyroid dysfunction | 22 (9.1) | | 21 (10.2) | 1 (2.9) | 0.282 |
| Smoking history | 128 (53.1) | | 112 (54.4) | 16 (45.7) | 0.444 |
|  |  | |  |  |  |
| LCPV | 36 (14.9) | | 30 (14.6) | 6 (17.1) | 0.889 |
| LAD | 41 [37, 46] | | 41 [37, 46] | 39 [34, 43] | 0.011 |
| LAVI | 39 [30, 47] | | 39 [31, 48] | 36 [23, 46] | 0.207 |
| LVEF | 57 [50, 63] | | 56 [50, 62] | 60 [54, 65] | 0.004 |
|  |  | |  |  |  |
| AAD | 99 (41.2) | | 89 (43.4) | 10 (28.6) | 0.144 |
| Class I AAD | 31 (12.9) | | 25 (12.1) | 6 (17.1) | 0.586 |
| Class III AAD | 72 (29.9) | | 67 (32.5) | 5 (14.3) | 0.048 |
| Digoxin | 3 (1.2) | | 2 (1.0) | 1 (2.9) | 0.916 |
| Verapamil | 2 (0.8) | | 2 (1.0) | 0 (0.0) | 1 |
| Betablocker | 172 (71.4) | | 147 (71.4) | 25 (71.4) | 1 |
|  |  | |  |  |  |
| Vit K Antagonist | 10 (4.5) | | 8 (4.3) | 2 (6.1) | 1 |
| DOAC | 210 (95.5) | | 179 (95.7) | 31 (93.9) | 1 |
|  |  | |  |  |  |
| Early recurrence | 48 (19.9) | | 38 (18.4) | 10 (28.6) | 0.247 |
| 1 month | 23 (47.9) | | 17 (44.7) | 6 (60) | 0.614 |
| Late recurrence 1y | 60 (24.9) | | 46 (22.3) | 14 (40.0) | 0.043 |

Table 1: Continuous variables expressed as ‘median [IQR]’ except for CHA2DS2-VA score ‘mean (SD)’, Factorial Variables as ‘absolute number (%)’. Abbreviations and Units: Age, years; BMI, Body mass Index, kg/m^2^; Persistent AF, Persistent Atrial fibrillation; CAD, Coronary Artery Disease; OSAS, Obstructive sleep apnoea syndrome; LCPV, Left common pulmonary vein; LAD, Left atrial diameter, mm; LAVI, left atrial volume index, ml/m2; LVEF, Left ventricular ejection fraction, %; AAD, Anti-Arhythmic Drugs; Vitamin K Antagonist (i.e. Marcoumar); DOAC, Direct oral anticoagulation; ILR, Implantable loop recorder; late recurrence, atrial arrhythmia of > 30 seconds duration after the first 3 months post-ablation.

## Table 2 Late recurrences in the 1^st^ year post ablation

|  | Overall | Holter | ILR |
| --- | --- | --- | --- |
| n | 60 | 46 | 14 |
|  |  |  |  |
| recurrence arrhythmia |  |  |  |
| AF | 53 (88) | 39 (85) | 14 (100) |
| AT | 5 (8) | 5 (11) | 0 |
| Flutter | 4 (7) | 4 (9) | 0 |
| Spontaneous resolved | 12 (20) | 7 (15) | 5 (36) |
| AAD therapy added/escalated | 18 (30) | 15 (33) | 3 (21) |
| BB | 9 (50) | 7 (47) | 2 (60) |
| Amiodarone | 9 (50) | 8 (53) | 1 (40) |
| Electrical cardioversion | 11 (18) | 10 (22) | 1 (7) |
| Ablation (EPS-RFA/ AV-node) | 6 (10) | 6 (13) | 0 |
| Repeat catheter ablation | 34 (57) | 26 (57) | 8 (57) |

Table 4: Factorial variables as ‘absolute number (%)’. Abbreviations: AF, atrial fibrillation; AT, atrial tachycardia; Flutter, atrial flutter; Spontaneous resolved, late recurrence disappeared without therapeutic intervention, such as electrical cardioversion or escalation of anti-arrhythmic therapy; AAD, anti-arrhythmic drug; BB, beta-blocker; Ablation (EPS-RFA/ AV-node), Electrophysiologic study with radiofrequency ablation or AV node ablation; Repeat catheter ablation, additional electrophysiologic study to treat late recurrence.

## Table 3 Predictors of LR in multivariate cox model

|  | HR | Lower 95CI | Higher 95CI |
| --- | --- | --- | --- |
| Age | 0.99 | 0.96 | 1.01 |
| Female | 1.57 | 0.91 | 2.7 |
| BMI | 1.00 | 0.95 | 1.07 |
| CHA2DS2-VA w/o age | 1.17 | 0.93 | 1.48 |
| Additional lesions | 0.74 | 0.42 | 1.31 |
| Persistent AF | 1.12 | 0.60 | 2.10 |
| ILR | 1.51 | 0.71 | 3.20 |
| LCPV | 0.68 | 0.32 | 1.45 |
| Early Recurrence 1 month | 3.56 | 1.76 | 7.05 |
| Early Recurrence 2/3 month | 6.07 | 3.25 | 11.33 |

Table S1 Abbreviations: BMI, Body mass Index; Persistent AF, Persistent Atrial fibrillation; ILR, Implantable loop recorder; LCPV, Left common pulmonary vein; late recurrence, atrial arrhythmia of > 30 seconds duration after the first 3 months post-ablation.

## Figure 1: Frequency of first atrial arrhythmia recurrence by month, irrespective of early or late classification.
